# Overt Primary Hypothyroidism in an Industrial Area in São Paulo, Brazil: The Impact of Public Disclosure

**DOI:** 10.3390/ijerph13111161

**Published:** 2016-11-22

**Authors:** Maria Angela Zaccarelli-Marino, Carmen Diva Saldiva André, Julio M. Singer

**Affiliations:** 1 Internal Medicine Department, Endocrinology Service, ABC Medical School Foundation, Santo André 09060-650, Brazil; 2 Department of Statistics, Institute of Mathematics and Statistics, University of São Paulo, São Paulo 05508-090, Brazil; carmensaldiva@gmail.com (C.D.S.A.); jmsinger@ime.usp.br (J.M.S.)

**Keywords:** autoimmune thyroiditis, industrial environment, petroleum byproducts, primary hypothyroidism

## Abstract

*Background*: Primary hypothyroidism (PH) is the most common thyroid pathology. *Purpose*: to evaluate the impact of public disclosure of an unexpected number of PH cases on the frequency of patients seeking medical evaluation for endocrinological diseases. *Methods*: data on 6306 subjects (3356 living in the surroundings of a petrochemical complex and 2950 in a control region) were collected over a 15-year time span. Thyroid function was determined by serum levels of triiodothyronine, thyroxine, free thyroxine and thyrotrophin. Antithyroglobulin and antithyroperoxidase antibodies and sonographic scans of the thyroid were performed in all patients. The data were analyzed via log-linear models to compute odds and odds ratios. *Results*: An increasing trend in the odds of PH was detected along the observation period with greater slope in the study region than in the control region. The odds of PH in the post-disclosure period (2002 to 2004) are greater than the corresponding ones in the pre-disclosure period (1989 to 2001). *Conclusions*: This study shows that living in the surroundings of a petrochemical complex may be an important risk factor for PH for both adults and children. Furthermore, public disclosure of such risk factor contributes to the awareness of the problem and to the possibility of an early diagnosis.

## 1. Introduction

In 1989, a patient diagnosed with overt primary hypothyroidism (PH), originated from a chronic autoimmune thyroiditis (CAT), called the attention of the first author of this study because he was a 37 year old male, working in a chemical industry and known to wash his hands with trichloroethylene, a volatile organic compound still used in industry as a degreasing agent. It is well known that trichloroethylene is highly toxic and considered as an important water and soil pollutant [1]. In adults, hypothyroidism can generate alterations of cardiac and cognitive functions, myxedema, hypercholesterolemia and incapacity to work or to lead a healthy family life [2,3,4,5]. In children, it may also have deleterious effects on growth, school performance and pubertal development. Adequate treatment leads to normal growth, puberty and normal final height [3,5,6]. Vanderpump et al. [7] emphasized that the cause of hypothyroidism is variable and depends on geographic and environmental factors as well as on genetic characteristics of the population and on age of disease onset.

Primary hypothyroidism is the most common thyroid pathology [8] and among other autoimmune thyroid diseases (AITD), CAT has been diagnosed with increased frequency in recent years [7,9]. In iodine sufficient regions, the major cause of PH is CAT [10]. According to Vanderpump et al. [7], patients with positive antithyroid antibodies are highly likely to develop hypothyroidism. These authors showed that 55% of the patients with elevated thyrotrophin (TSH) and positive antithyroid antibodies develop hypothyroidism in contrast to 33% of the patients who have only elevated TSH with negative antithyroid antibodies.

Benvenga and Trimarchi [11] reported that Hashimoto’s thyroiditis (HT) has become 10 times more common than it was since the early 1990s with males being relatively more represented; they also showed that only environmental changes, as opposed to genetic changes, can account for such alterations in the presentation of HT. Rizzo et al. [12] also mention that only environmental modifications can explain the great increase in the annual frequency of HT that occurred in such a relatively short period of time.

According to Gilbert et al. [13], certain chemical agents like trichloroethylene may induce an autoimmune response in rats. These authors conjecture that suppression of immune responses in rodents may be predictive of a similar response in humans and that there is a relationship between immune suppression following developmental exposure to toxicants and enhanced risk of autoimmune or neoplastic disease [14]. Nineteen years before, Bahn et al. [15] had already observed an increased incidence of antimicrosomal thyroid antibodies and hypothyroidism in male factory workers exposed to other chemical agents, like polyhalogenated biphenyls (PBB) and polyhalogenated biphenyl oxides. Thirty years later, Brent [16] showed that environmental exposure to a series of chemicals, ranging from perchlorate to polychlorinated biphenols may affect thyroid function. This author concluded that most of these agents are associated with reduced thyroid hormone levels or impaired thyroid action and that environmental exposure to such agents induce an increased risk of autoimmune thyroid disease. Other authors [17] observed that organic pollutants such as polyaromatic hydrocarbons are associated with goiter and thyroid disease. On the other hand, Yang et al. [18] and Neuberger et al. [19] showed that cancer prevalence is higher for individuals living in areas surrounding plants that manufacture petroleum byproducts.

In 2002, the large number of cases of PH originated from CAT detected in patients seeking medical care in a densely populated area in the vicinity of a petrochemical complex was communicated by this first author to the Municipal and State Public Health Services and to the National Health Foundation (FUNASA) and was publicly disclosed. The frequency of patients with the same diagnosis increased between 2002 and 2004.

The objective of this study is to evaluate the impact of public disclosure of an unexpected number of PH on the frequency of patients seeking medical evaluation for endocrinological diseases. This is accomplished by comparing the odds of occurrence of PH in children and adults of both sexes living in the surroundings of a petrochemical complex seeking medical care with the corresponding odds for a population living in the vicinity of a steel producing industrial complex before and after the notification of the abnormal number of cases to public health authorities in 2002.

The data were collected over a 15-year time span, from 1989 to 2004 in two regions. The first (Region A), located on the boundary of the municipalities of Santo André, Mauá and São Paulo, state of São Paulo (SP), Brazil, consists of a densely populated area of 125 ha in the vicinity of large industrial plants that manufacture petroleum byproducts including polyethylene and polypropylene from naphtha distillation as well as various intermediate substances that are used as raw materials for manufacturing other products. The second (Region B) is a steel producing industrial area located on the boundary of the municipalities of Santo André, São Bernardo do Campo and São Caetano do Sul, SP, Brazil, about 8.5 km away from Region A. No plants that manufacture petroleum byproducts are located in this region, treated as a control region in this study. The state environmental agency (CETESB) has a continuous air quality monitoring station in this area, where only the levels of particulate matter (PM_10_) and ozone (O_3_) are recorded. This study is a sequel to a survey about CAT in industrial areas in Brazil [20].

## 2. Methods

We compare two periods: the first consisting of 12 years (1989 to 2001), when the patients living in Region A spontaneously searched an endocrinology clinic located in Santo André, SP, Brazil and the second consisting of a 3 year period (2002 to 2004), starting after the first author notified the Epidemiological Surveillance Center (ESC) of the Department of Health of the State of São Paulo regarding the excessive number of PH observed in her endocrinology clinic.

### 2.1. Ethical Statement

This research was approved by the Committee of Ethics in Research of the Medical School of the ABC Foundation, SP, Brazil and registered under number 284/2007. The objectives and methods of this study were clearly stated to all patients or to parents in the case of children. All patients agreed to participate in this study.

### 2.2. Subjects

The 6306 subjects (3356 in Region A and 2950 in Region B) included in the study were selected from patients seeking medical attention and general endocrinological evaluation at an endocrinology clinic located in Santo André, SP, Brazil, run by the first author (Maria Angela Zaccarelli-Marino). Clinical history and physical examination including weight and height measurements, conducted by Maria Angela Zaccarelli-Marino, were performed in all 6306 patients selected for this study. Patients aged 18 or more were considered as adults and those aged 17 or less, as children. Only patients living in the same region for 10 years or more were included in the study. Furthermore, controls (patients from Region B) were matched with those from Region A according to social and educational status. Patients taking medications that could interfere with the outcome, including amiodarone, lithium carbonate, iodine and interferon, as well as those with hyperthyroidism, malignant nodules, previous thyroid treatment with radioiodine or radiotherapy of the chest or neck, primary hyperthyroidism of autoimmune origin (antibody anti-TSHR positive) were excluded from the study. Patients that showed nodular goiter, with nodules greater than 1 cm, were referred for fine needle aspiration cytology, and those diagnosed with carcinoma were referred to surgery and excluded from the study. Employees in the chemical industries or those connected to agriculture in contact with pesticides were excluded from the study.

### 2.3. Sample Collection

Blood samples were collected, at a private laboratory (Fleming Medicina Diagnóstica) in the municipality of Santo André, SP. All patients spontaneously came to the laboratory, in the morning after 8 h of fasting. The children came accompanied by their parents or responsible ones. The blood samples were obtained by standard techniques. The collected blood was placed in adequate tubes and analyzed by standard procedures. Dosages of triiodothyronine (T3), thyroxine (T4), free thyroxine (FT4), thyrotrophin (TSH), antithyroglobulin antibody (A-Tg) and A-TPO (antithyroperoxidase antibody) were performed at the same laboratory using chemiluminescence (closed system, Immulite, DPC-MedLab, Los Angeles, CA, USA) or electrochemiluminescence (Ellecsys, Roche, Basel, Switzerland).

### 2.4. Thyroid Hormones Measurement

Thyroid function was determined by basal serum hormone levels of total T3, total T4, FT4 and TSH, obtained by chemiluminescence and electrochemiluminescence. The levels of FT4 were obtained only for patients examined after 1994.

Serum levels of A-Tg were obtained by fluoroimmunoassay and considered positive above 40 U/mL or by a chemiluminescence technique (also considered positive above 40 IU/mL). Serum levels of A-TPO were obtained by radioimmunoassay (considered positive above 60 U/mL) or by a chemiluminescence technique (considered positive above 35 IU/mL).

### 2.5. Sonographic Scan

Sonographic scans of the thyroid gland were performed by the same physicians with ample experience in image diagnosis (DISA) in the municipality of Santo André, SP using echography with a high resolution multi-frequency linear transducer (7.5 MHz and 10 MHz). Scans that showed homogeneous echotexture and a thyroid volume (ThV) between 6.0 and 17.1 cm^3^ were considered normal.

CAT was diagnosed in patients who were positive for antibodies to thyroglobulin and to thyroperoxidase and who showed heterogeneous texture and marked hypoechogenicity in the sonographic scans of their thyroid glands. Tests for A-Tg and A-TPO were performed because in iodine sufficient regions, CAT is the major cause of PH [10]. Iodine sufficiency for Region A was demonstrated in previous studies [21,22,23]. All patients selected for this study were diagnosed with CAT and all patients were positive for antibodies to thyroglobulin and to thyroperoxidase.

Overt PH was diagnosed when signs and clinical symptoms were present along with low total T4 (chemiluminescence, with normal values for children and adolescents between 6.0–13.5 µg/dL and adults between 4.5–12.5 µg/dL or electrochemiluminescence, with normal values for children and adolescents between 7.6–13.4 µg/dL and adults between 5.1–14.1 µg/dL), low FT4 (chemiluminescence with normal values for children, adolescents and adults between 0.8–1.9 ng/100 mL or electrochemiluminescence with normal values for children and adolescents between 1.1–1.8 ng/dL and adults between 0.9–1.7 ng/dL), TSH ≥ 10 µIU/mL [9] (chemiluminescence with normal values for children, adolescents and adults between 0.4–4.0 µIU/mL or electrochemiluminescence with normal values for children, adolescents and adults between 0.2–8.0 µIU/mL) and low total T3 (chemiluminescence with normal values for children, adolescents and adults between 70–170 ng/dL or electrochemiluminescence with normal values for children and adolescents between 80–260 ng/dL and adults between 80–200 ng/dL). All patients diagnosed with PH in this study have been treated with sodium levothyroxin. 

### 2.6. Statistical Analysis

Initially, the odds of PH for each combination of the levels of age and sex in regions A and B were estimated in (approximately) three year periods starting in 1989; corresponding 95% confidence intervals were also constructed. The same technique was applied to estimate the odds ratio of PH between the two periods of interest (after public disclosure (2002 to 2004) and before public disclosure (1989 to 2001)) for each region, age and sex. Similarly, the odds ratio for PH between the two regions (A and B) in each period (before and after public disclosure) for each age and sex was also computed.

The confidence interval limits for the odds or for the odds ratios were obtained by exponentiation of the corresponding limits of confidence intervals for their logarithms to achieve a better approximation by the Gaussian distribution. The odds of PH for the entire period 1989 to 2001 were computed as the geometric mean of the corresponding triennial odds. Wald chi-squared tests were used to detect significant odds ratios of PH between periods within regions or regions within periods. Details may be obtained in Agresti [24], for example. Computations were performed via the log-linear functions in the ACD (Analysis of Categorical Data) library available in the R (R Foundation for Statistical Computing, Vienna, Austria) statistical analysis package [25] and the codes may be obtained from the authors upon request.

## 3. Results

The percentage of patients with PH classified by sex, age and region and grouped in periods of approximately three years (to facilitate comparison of pre and post disclosure periods) are displayed in Table 1.

**Figure 1:**
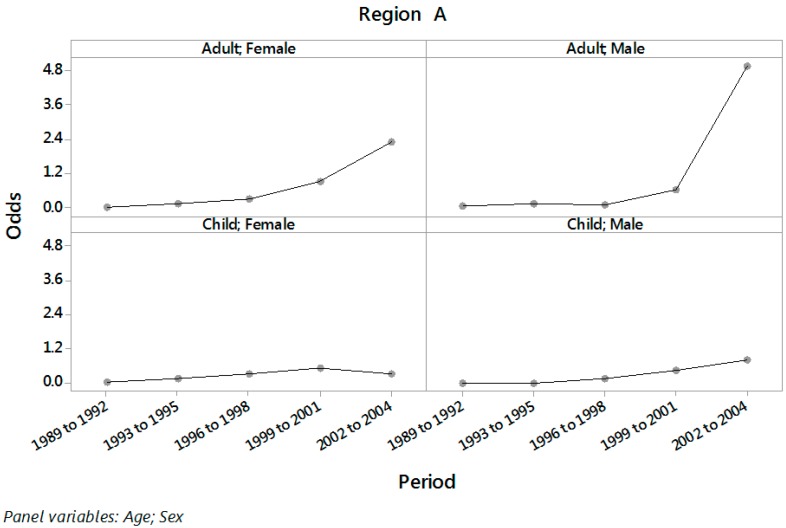
Odds of PH in periods from 1989 to 1992, 1993 to 1995, 1996 to 1998, 1999 to 2001 and 2002 to 2004 for each combination of age and sex in Region A.

**Figure 2:**
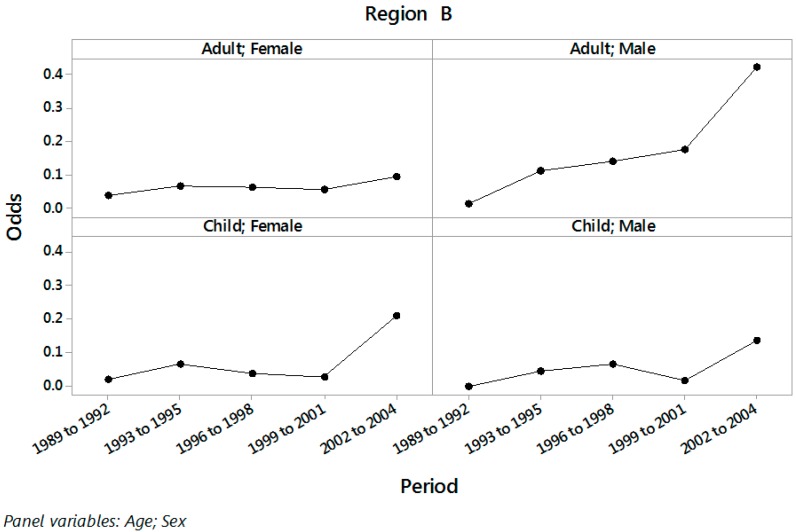
Odds of PH in periods from 1989 to 1992, 1993 to 1995, 1996 to 1998, 1999 to 2001 and 2002 to 2004 for each combination of age and sex in Region B.

Plots of the odds of PH in Regions A and B (by sex and age) for the different three year periods are displayed in Figure 1 and Figure 2.

An increasing trend in the odds of PH along the triennial periods is observed in these figures, with larger slope in Region A than in Region B and for adults than for children. The estimated odds of PH along with 95% confidence intervals for each combination of the levels of age and sex in regions A and B for the periods 1989 to 2001 (before public disclosure) and 2002 to 2004 (after public disclosure) are displayed in Table 2.

The data in Table 2 indicate that for both study periods, the odds of PH in Region A are greater than those in Region B, whatever the age or sex of the participants. Accordingly, for participants of the same age and sex, the odds of PH in the post-disclosure period (2002 to 2004) are greater than the corresponding ones in the pre-disclosure period (1989 to 2001).

The odds ratio of PH between the two periods ((2002 to 2004) and (1989 to 2001)) in each region, age and sex and, as well as the corresponding odds ratio between the two regions (A/B) in each period (before and after public disclosure) for each age and sex, along with 95% confidence intervals are presented in Table 3 and Table 4. The results displayed in Table 3 show that the odds of PH in the 2002 to 2004 period are significantly greater than the corresponding odds in the 1989 to 2001 period in either region and for all combinations of the categories of age and sex. From Table 4 we may conclude that there is no significant difference between the odds of PH for the two regions for: (a) male children and male adults in the 1989 to 2001 period and (b) female children in the 2002 to 2004 period. Otherwise, the odds of PH in Region A are significantly greater than those in Region B.

## 4. Discussion

Our results indicated that the odds of PH for residents in the vicinity of petrochemical plants that manufacture polyethylene, polypropylene and many other products (Region A) are larger than those for residents in a control region (Region B) and have an increasing trend over the study period (1989 to 2004) for both children and adults of either sex. Furthermore, the results suggested that the odds of PH increase drastically after public disclosure of an abnormal frequency of the disease. We conjecture that such environmental contaminants might be a cause of the unusual number of thyroid pathology detected.

Landrigan et al. [26] showed that environmental pollutants, chemicals, solid waste and pharmaceuticals have a negative impact on the developing immune system of children and this may justify the increased odds of PH in Region A reported for children since all the children diagnosed with PH were also diagnosed with CAT.

Radetti et al. [27], in a multicentre study, investigated the outcome of euthyroid children with HT and showed that 64.8% of them remained euthyroid, 9.5% progressed to subclinical hypothyroidism and 25.7% to overt hypothyroidism after 5 years. Although we are not evaluating prevalence, the larger odds of PH observed for males suggest that the prevalence of PH in Region A is greater than those in Vanderpump and Tunbridge [28] who reported a prevalence of 2% in an adult female population as opposed to 0.2% in a male population. In fact, the prevalence of PH in different adult populations are reported in many studies [7,28,29,30,31,32] and range from 0.1% to 2.0%, but not much is known about the corresponding prevalence in children.

Benvenga et al. [33] have demonstrated that in most industrialized countries, autoimmune disorders including chronic lymphocytic thyroiditis (CLT) are increasing. This increase parallels the one regarding differentiated thyroid cancer and these thyroid diseases could be related by certain environmental factors, such as polluting substances acting as endocrine disrupting chemicals. Arena et al. [34] concluded that the petrochemical complex-related pollutions in an environmental factor involved in the development of CLT and, likely, in the CLT association with thyroid neoplasms.

The concentration of iodine has been considered a modulating factor for thyroid autoimmunity [35]. Zak et al. [36], for example, observed a decrease in the frequency of new cases of CLT from 30% in 1999 to 10% in 2004, probably because of the introduction of mandatory iodine prophylaxis in Poland in 1997. Langer et al. [37], on the other hand, found that the levels of urinary iodine were within normal limits in the residents of a polluted area. A populational study conducted in China showed that an augmented ingestion of iodine may be considered as a risk factor for the progression of PH [38]. Although hypothyroidism appears to be common among the adult population of Isfahan, Iran, its high prevalence is probably due to autoimmunity, with no correlation to iodine intake [39].

The major cause of PH in iodine sufficient regions is CAT [10] and although the presence of A-TPO defines the diagnosis of CAT, all the 1078 children and adults (905 in Region A and 173 in Region B) with PH evaluated in this study (Table 1) presented A-Tg and A-TPO positive antibodies; also, the sonographic scans of their thyroid glands showed heterogeneous texture and marked hypoechogenicity.

In Brazil, Rosario et al. [40] have shown that not only the presence of A-TPO antibodies, but also that ultrasonographic aspects of CAT are associated with a greater risk of progression to PH. Marcocci et al. [41] concluded that patients with CAT and hypoecogenicity in thyroid ultrassonographic exams have a greater probability to progress to PH. In fact, Huber et al. [42] showed that the annual incidence of PH in the presence of antithyroid antibodies is 11.4%.

A few Brazilian studies [22,43,44] have shown iodine sufficiency in the general population and a study conducted in Region A [21] also came to a similar conclusion. The average urine iodine was 26.5 μg/dL (reference values ranging from 10–30 μg/dL) in Region A and iodine concentration in table salt consumed in the same region was 31.9 mg/kg which lies within the acceptable limits of 20–60 mg/kg, suggesting that environmental factors are the most plausible cause of the reported results of our study.

According to Brent [16], although approximately 70% of the risk for developing autoimmune thyroid disease is attributable to genetic background, environmental triggers are thought to play a role in this process for susceptible individuals.

Sgarbi and Maciel [45] also reported that the interaction between genetic susceptibility and environmental factors appears to be of fundamental importance to initiate the process of thyroid autoimmunity. No significant differences between the average concentrations of particulate matter (PM_10_) and ozone (O_3_) in the two regions considered in this study were identified.

In our study, PH and CAT were diagnosed in residents coming from different families including married couples thus circumventing the influence of genetic factors. In fact, our conjectures regarding the high prevalence of CAT in Region A was corroborated in a study sponsored by the Epidemiological Surveillance Center (ESC) of the Department of Health of the State of São Paulo [21].

Certain pharmacologic agents like interferon, amiodarone, iodine and lithium may also cause autoimmune thyroid diseases and PH in predisposed individuals [46]. Patients taking these medications were excluded from our study; this may be considered as further evidence that the vicinity to the petrochemical complex is the most probable cause of the abnormal cases of PH detected in our study.

Finally, we observed that the odds of PH in children and adults of both sexes in Region A increased drastically after the public health authorities were notified in 2002. This observation suggests that awareness of the population contributed to new diagnoses of PH and before that the signs and symptoms of hypothyroidism were not taken into account, mainly in children with learning difficulties, memory problem, attention and concentration problem and growth deficits.

The patients with clinical and laboratory PH were treated with sodium levothyroxine and remain with normal thyroid function; however the A-Tg and A-TPO of these patients are still above normality. This suggests that there is no treatment for CAT, thus opening a new field for research.

Given the observational nature of our study, it is subject to many limitations, among which we mention the lack of objective control of confounding factors like social and educational status of the participants or quantification of actual exposure to specific organochlorinated products such as pesticides and solvents. A follow up collaborative study with the Laboratory of Atmospheric Pollution of the University of São Paulo Medical School is already being conducted.

## 5. Conclusions

We detected an increasing trend in the odds of PH along the observation period with greater slope in the study region than in the control region. We also noted that the odds of PH in the post-disclosure period (2002 to 2004) are greater than the corresponding ones in the pre-disclosure period (1989 to 2001). After the 2002 public disclosure of this increase in the frequency of PH, the information disseminated by health service professionals and teachers in schools contributed to the awareness of the problem leading to an early diagnosis, especially in children. Given the asymptomatic characteristic of the disease in its initial development individuals and the risk of its complications, our suggestion is that the thyroid gland must be evaluated in employees of chemical plants as well as in residents living around such industrial areas.

## Figures and Tables

**Table 1:** Percentage of patients with primary hypothyroidism (PH) in regions A and B by period, age and sex.

Period	Age	Sex	Region A		Region B	
			Number of Patients	% of Patients with PH	Number of Patients	% of Patients with PH
1989 to 1992	Adult	Male	83	2.4	75	1.3
		Female	572	0.5	550	3.6
	Child	Male	43	0.0	62	0.0
		Female	85	2.4	107	1.9
1993 to 1995	Adult	Male	58	8.6	49	10.2
		Female	466	8.8	411	6.1
	Child	Male	21	0.0	45	4.4
		Female	57	12.3	98	6.1
1996 to 1998	Adult	Male	116	6.0	65	12.3
		Female	454	22.2	430	6.0
	Child	Male	28	14.3	48	6.3
		Female	54	24.1	81	3.7
1999 to 2001	Adult	Male	71	38.0	54	14.8
		Female	417	47.5	399	5.3
	Child	Male	37	29.7	64	1.6
		Female	43	34.9	71	2.8
2002 to 2004	Adult	Male	149	83.2	27	29.6
		Female	429	69.5	241	8.7
	Child	Male	29	44.8	33	12.1
		Female	144	23.6	40	17.5

**Table 2:** Odds of primary hypothyroidism (PH) and 95% confidence intervals (CI) for the pre-public disclosure period (1989 to 2001) and odds of PH and 95% confidence intervals (CI) for the post-public disclosure period (2002 to 2004).

Period	Age	Sex	Region A		Region B	
			Odds	CI	Odds	CI
1989 to 2001	Child	Female	0.155	(0.098; 0.245)	0.034	(0.019; 0.063)
		Male	0.066	(0.024; 0.188)	0.025	(0.010; 0.066)
	Adult	Female	0.107	(0.079; 0.145)	0.054	(0.044; 0.067)
		Male	0.098	(0.061; 0.158)	0.078	(0.043; 0.143)
2002 to 2004	Child	Female	0.309	(0.210; 0.454)	0.212	(0.094; 0.480)
		Male	0.813	(0.391; 1.689)	0.138	(0.048; 0.392)
	Adult	Female	2.275	(1.852; 2.794)	0.095	(0.061; 0.149)
		Male	4.960	(3.227; 7.623)	0.421	(0.184; 0.962)

The odds of PH for the period 1989 to 2001 (for each combination of the levels of region, sex and age) were computed as the geometric mean of the corresponding triennial odds obtained from Table 1. The standard errors used to construct the approximate confidence intervals were computed via the log-linear functions available in the library ACD (Analysis of Categorical Data) in the software package R.

**Table 3:** Odds ratios of primary hypothyroidism and 95% confidence intervals (CI) for comparison between the post public disclosure period (2002 to 2004) and the pre public disclosure period (1989 to 2001).

Age	Sex	Region A			Region B		
		Odds Ratio	CI	*p*	Odds Ratio	CI	*p*
Child	Female	2.00	(1.10; 3.64)	0.024	6.18	(2.23; 17.12)	<0.001
	Male	12.22	(3.43; 43.55)	<0.001	5.49	(1.32; 22.86)	0.019
Adult	Female	21.24	(14.72; 30.65)	<0.001	1.76	(1.07; 2.88)	0.026
	Male	50.67	(26.69; 96.21)	<0.001	5.38	(1.93; 14.98)	0.001

Each odds ratio in Table 3 (i.e., for each combination of the levels of sex and age) was obtained as the quotient between the corresponding odds for the period 2002 to 2004 and the odds for the period 1989 to 2001 available in Table 2. The standard errors used to construct the approximate confidence intervals were computed via the log-linear functions available in the library ACD in the software package R.

**Table 4:** Odds ratios of primary hypothyroidism and 95% confidence intervals (CI) for comparison between Region A and Region B.

Age	Sex	1989 to 2001			2002 to 2004		
		Odds Ratio	CI	*p*	Odds Ratio	CI	*p*
Child	Female	4.54	(2.10; 9.66)	<0.001	1.46	(0.59; 3.59)	0.413
	Male	2.65	(0.64; 10.96)	0.179	5.89	(1.64; 21.10)	0.007
Adult	Female	1.97	(1.36; 2.85)	<0.001	23.83	(14.56; 39.00)	<0.001
	Male	1.25	(0.58; 2.70)	0.570	11.78	(4.64; 29.89)	<0.001

Each odds ratio in Table 4 (i.e., for each combination of the levels of sex and age) was obtained as the quotient between the corresponding odds for Region A and the odds for Region B available in Table 2. The standard errors used to construct the approximate confidence intervals were computed via the log-linear functions available in the library ACD in the software package R.
